# *Blastosporiumpersicolor* gen. et sp. nov., a new helotialean fungus

**DOI:** 10.3897/mycokeys.51.30798

**Published:** 2019-04-22

**Authors:** Hua Zheng, ZhenNa Zhang, ZhiJia Wen, Rafael F. Castañeda-Ruiz, ZeFen Yu

**Affiliations:** 1 Laboratory for Conservation and Utilization of Bio-resources, Key Laboratory for Microbial Resources of the Ministry of Education, Yunnan University, Kunming, Yunnan, 650091, China; 2 Xiamen Tobacco Industrial CO., LTD, Xiamen, Fujian, China; 3 Associate Research of Instituto de Investigaciones Fundamentales en Agricultura Tropical Alejandro de Humboldt (INIFAT), Calle 1 Esq. 2, Santiago de Las Vegas, C. Habana, C.P. 17200, Cuba

**Keywords:** Ascomycota, Pezizomycotina, phylogeny, *
Nicotiana tabacum
*

## Abstract

A new genus and species, *Blastosporiumpersicolor*, is described and illustrated from leaves of mildewed tobacco. It is characterised by branched, septate hyphae from which arise macronematous, unbranched or spaced branched conidiophores and mono- or polyblastic conidiogenous cells that produced solitary and blastocatenate, obovoid, oblong, ellipsoidal, allantoid, broad fusiform to irregular, unicellular, hyaline conidia. The phylogenetic analyses, based on the combined sequence data from the small and large nuclear subunit ribosomal DNA (SSU and LSU), placed *B.persicolor* in the Leotiomycetes class, Helotiales order.

## Introduction

The Kingdom Fungi contains a huge number of species, which continues to rise with more collections. With the advance in the studies of DNA sequence data, the fungal classification system has been updated over the years. Many described species obtained new taxonomic status after the molecular data and have been processed. Leotiomycetes is a large class in Ascomycota and has potential taxonomic value relating to the ecology and biology. The traditional classification of Leotiomycetes at high levels has experienced considerable challenges with the inclusion of the molecular techniques in systematics studies. For example, early research accepted five orders, 21 families and about 510 genera in the Leotiomycetes on the basis of both traditional classification and molecular phylogenetic studies (Eriksson 2005, Kirk et al. 2001), but a recent study reported a new classification of Leotiomycetes, including 11 orders, 44 families and about 590 genera (Wijayawardene et al. 2018) and this classification also lacks sufficient DNA sequence data. In Leotiomycetes, the order Helotiales, one of the largest non-lichen-forming ascomycetous groups, is composed of fungi of diverse morphology and ecology. Of these, members of the Helotiales thrive in various ecosystems and cover a broad range of niches and helotialean fungi have been found as plant pathogens, endophytes, nematode-trapping fungi, mycorrhizae, ectomycorrhizal parasites, fungal parasites, terrestrial saprobes, aquatic saprobes, root symbionts and wood rot fungi (Wang et al. 2006).

During a survey of fungi growing on mildewed tobacco leaves, an unknown fungus was found. Based on its morphological characters and DNA sequence data, it is proposed as a new asexual genus and species, *Blastosporiumpersicolor*.

## Materials and methods

### Isolation and morphological study of strain

Samples of the mildewed tobacco leaves were collected from Xiamen Logistics Warehousing Center. Samples were preserved in zip-locked plastic bags, labelled and transported to the laboratory. The procedure was as follows: samples (5g) were placed in PDA liquid medium (200 g potato, 20 g glucose, 1000 ml distilled water), shaken at 140 rpm/min for 1 h and the filtrate was collected. The filtrate was coated on a CMA plate (20 g cornmeal, 10 g agar, 1000 ml distilled water) at 28 °C, supplemented with two antibiotics (penicillin G, 0.5 g/l; and streptomycin, 0.5 g/l; Gams et al. 1998). After 3–5 days, single colonies were isolated into pure culture, grown on potato dextrose agar plates (PDA). The characteristics of the colonies were from PDA, CMA and SNA (synthetic low nutrient agar). Microscopic characteristics were made from cultures growing on CMA after incubation at room temperature for one week.

The pure cultures and dried cultures were deposited in the Herbarium of the Laboratory for Conservation and Utilization of Bio-resources, Yunnan University, Kunming, Yunnan, P.R. China (YMF, formerly Key Laboratory of Industrial Microbiology and Fermentation Technology of Yunnan).

### *DNA extraction, polymerase chain reaction (PCR) amplification and sequencing*

Pure cultures were grown on PDA for 5 days at 25 °C. Actively growing mycelium was scraped off the surface of a culture and transferred to 2 ml Eppendorf micro-centrifuge tubes. Total genomic DNA was extracted according to the procedures in Turner et al. (1997). Primers used for PCR amplification and sequencing of nucSSU rDNA, nucLSU rDNA and ITS rDNA were NS1-NS4, LROR-LR7 and ITS1-ITS4, respectively (White et al. 1990, Vilgalys and Hester 1990). Detailed protocols and PCR conditions for the amplification were fully described by Su et al. (2015). PCR products were then purified using a commercial Kit (Bioteke Biotechnology Co, Ltd, China) and forward and reverse sequences with a LI-COR 4000L automatic sequencer, using a Thermo Sequenase-kit, as described by Kindermann et al. (1998). The sequences were deposited in the National Center for Biotechnology Information (NCBI) and the accession numbers are listed in Table 1.

**Table 1.** Strains and the GenBank accession numbers of sequences used in the molecular phylogenetic analyses in this study.

Name	Strain	GenBank accession number	
		LSU	SSU
*Arthoniacaesia* (Flot) Körb.	AFTOL-ID 775	FJ469668	–
*Arthrobotryselegans* (Subram & Chandrash) Seifert & W.B. Kendr.	AFTOL-ID 1252	FJ176864	FJ176810
*Arthrocladiellamougeotii* (Lév) Vassilkov	–	AB022379	AB033477
*Blastosporiumpersicolor* Z. F. Yu & H. Zheng	**YMF1.05546**	**MH992517**	**MH992516**
*Blumeriagraminis* (DC.) Speer	–	AB022362	AB033476
*Brasiliomycestrinus* (Harkn) R.Y. Zheng	–	AB022350	–
*Bryoglossumgracile* (P. Karst.) Redhead	MBH52481	AY789420	AY789419
*Bulgariainquinans* (Pers.) Fr.	ZW-Geo52-Clark	AY789344	AY789343
*Candidaalbicans* (C.P. Robin) Berkhout	WO1	L28817	X53497
*Capnodiumcoffeae* Pat.	CBS 147.52	DQ247800	DQ247808
*Chlamydotubeufiahuaikangplaensis* Boonmee & K.D. Hyde	MFLUCC10-0926	JN865198	–
*Ciboriabatschiana* (Zopf) N. F. Buchw.	WZ-JXD-22	AY789322	–
*Cudoniacircinans* (Pers.) Fr.	OSC56399	AF279379	AF107343
*Cyttariadarwinii* Berk.	14	EU107208	EU107181
*Dermeaacerina* (Peck) Rehm	CBS 161.38	DQ247801	DQ247809
*Disciotisvenosa* (Pers.) Arnould	AFTOL-ID 179	AY544667	AY544711
*Dothideasambuci* (Pers.) Fr.	AFTOL-ID 274	AY544681	AY544722
*Erysipheaustraliana* (McAlpine) U. Braun & S. Takam.	–	AB022407	–
*Erysiphecornicola* Meeboon & S. Takam.	–	AB022389	–
*Erysipheglycines* F. L. Tai	MUMH52	AB022397	AB120748
*Erysiphegracilis* R. Y. Zheng & G. Q. Chen	–	AB022357	–
*Erysiphemori* (I. Miyake) U. Braun & S. Takam.	–	AB022418	AB033484
*Erysiphesimulans* (E. S. Salmon) U. Braun & S. Takam.	–	AB022395	–
*Eupenicilliumlimosum* S. Ueda	AFTOL-ID 2014	EF411064	EF411061
*Fabrellatsugae* (Farl) Kirschst.	–	AF356694	–
*Geoglossumglabrum* Pers.	OSC60610	AY789317	AY789316
*Geoglossumumbratile* Sacc.	Mycorec1840	AY789303	AY789302
*Helicomachlamydosporum* Shearer	CBS 160.69	AY856875	AY856923
*Helicomavaccinii* Carris	CBS 216.90	AY856879	AY856926
*Helicomycesroseus* Link	CBS 283.51	AY856881	AY856928
*Helicosporiumguianense* Linder	CBS 269.52	AY856893	AY856938
*Holwayamucida* (Schulzer) Korf & Abawi	B 70 0009352	DQ257356	DQ257355
*Lachnumbicolor* (Bull.) P. Karst.	AFTOL-ID 177	AY544674	AY544690
*Lachnumvirgineum* (Batsch) P. Karst.	AFTOL-ID 49	AY544646	AY544688
*Leotialubrica* (Scop.) Pers.	ZW-Geo59-Clark	AY789359	AY789358
*Monascuspurpureus* Went	AFTOL-ID 426	DQ782908	DQ782881
*Morchellaesculenta* (L.) Pers.	AFTOL-ID 60	AY544664	AY544708
*Mycosphaerellapunctiformis* (Pers.) Starbäck	AFTOL-ID 942	DQ470968	DQ471017
*Neoerysiphegaleopsidis* (DC.) U. Braun	–	AB022369	–
*Neofabraeamalicorticis* (Cordley) H.S. Jacks.	AFTOL-ID 149	AY544662	AY544706
*Orbiliavinosa* (Alb. & Schwein.) P. Karst.	AFTOL-ID 905	DQ470952	DQ471000
*Penicilliumfreii* Frisvad & Samson	DAMO 216705	AY640958	AY640998
*Phyllactiniamoricola* (Henn.) Homma	–	AB022401	AB033481
*Piceomphalebulgarioides* (P. Karst.) Svrček	1589.P	Z81415	–
*Pleochaetashiraiana* (Henn.) Kimbr. & Korf	MUMH36	AB022403	AB120750
*Podosphaeratridactyla* (Wallr.) de Bary	–	AB022393	–
*Roccellographacretacea* J. Steiner	AFTOL-ID 93	DQ883696	DQ883705
*Rutstroemiabolaris* (Batsch) Rehm	1526.P	Z81419.1	–
*Sawadaeapolyfida* (C.T. Wei) R.Y. Zheng & G.Q. Chen	–	AB022364	–
*Schismatommadecolorans* (Erichsen) Clauzade & Vězda	DUKE 0047570	NG_027622	NG_013155
*Scleromitrulashiraiana* (Henn.) S. Imai	Hirayama062001	AY789407	AY789406
*Sclerotiniasclerotiorum* (Lib.) de Bary	WZ0067	AY789347	AY789346
*Spathulariaflavida* Pers.	wz138	AF433142	AY789356
*Thaxteriellainthanonensis* Boonmee & K.D. Hyde	MFLUCC11-0003	JN865199	–
*Trichoglossumhirsutum* (Pers.) Boud.	AFTOL-ID 64	AY789313	AY789312
*Vibrisseaflavovirens* (Pers.) Korf & J.R. Dixon	MBH39316	AY789426	AY789425
*Vibrisseatruncorum* (Alb. & Schwein) Fr.	CUP-62562	AY789402	AY789401

### Sequence alignment and phylogenetic analysis

Other fungal sequences were obtained from the GenBank nucleotide database. DNA sequence data were aligned using ClustalX 1.83 (Higgins 1994) with default parameters and the consensus sequences were manually adjusted and linked in BioEdit v.7.0 (Hall 1999). Manual gap adjustments were made to improve the alignment and ambiguously aligned regions were also excluded. Portions of the 5'- and 3'-ends of the nuclear small and large subunits ribosomal DNA (nucSSU and nucLSU) were excluded from all analyses and coded by a question mark (?). MrBayes (Ronquist and Huelsenbeck 2003) was used to calculate the SSU rRNA and LSU rRNA sequence-based Bayesian inference of the phylogeny tree, with the following parameters: ngen=1,000,000; samplefr=1,000; printfr=1,000. The GenBank accession numbers of sequences used in the phylogenetic analysis are shown in Table 1 including the classes of Leotiomycetes, Arthoniomycetes, Dothideomycetes, Eurotiomycetes, Orbiliomycetes, Pezizomycetes and Sordariomycetes. *Candidaalbicans* (C.P. Robin) Berkhout (Saccharomycetes) was used as outgroup.

## Results

### *Sequence analyses*

**Figure 1.**
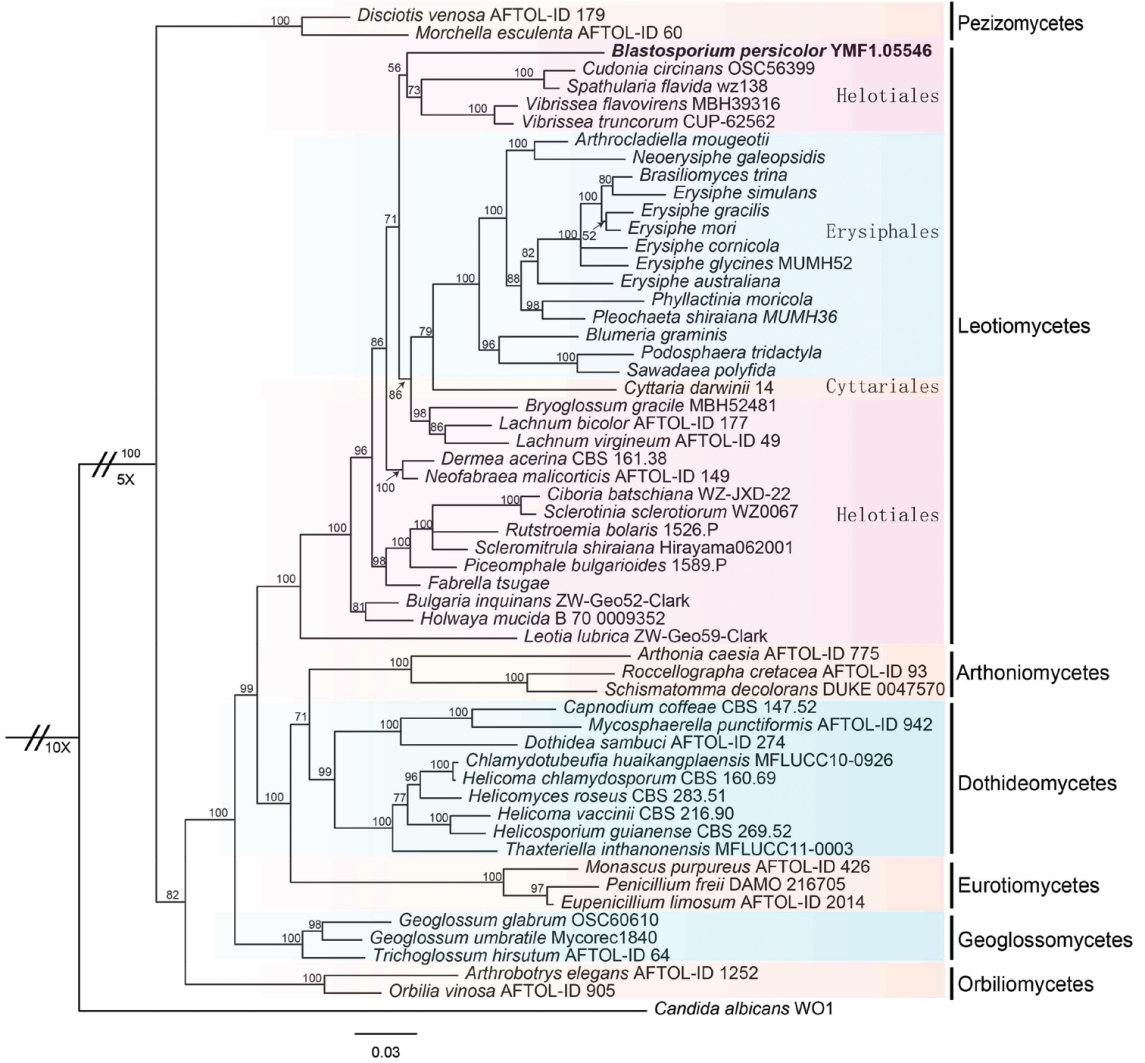
Phylogenetic tree based on Bayesian analysis of the combined LSU and SSU sequences. *Candidaalbicans* is used as outgroup. Bayesian bootstraps were indicated by the nodes and the scale bar shows the expected changes per site. The new genus proposed is in boldface.

In BLAST searches, the ITS sequence *B.persicolor*, MH992518, had the highest similarity of 88% with *Tetracladium* and 87% with *Chalara* (Corda) Rabenh., both belonging to Leotiomycetes. Therefore, most sequences are mainly from Leotiomycetes in the dataset. The dataset comprised 57 taxa representing 7 classes, 11 orders, 22 families and 57 species with *Candidaalbicans* as outgroup. Other DNA sequences were obtained from the GenBank. The final alignment comprised a total of 1635 base pairs (TreeBASE accession number: 23451), which combined the SSU rRNA and LSU rRNA sequences and the dataset was analysed by the Bayesian Inference method. The topologies of the tree are shown with the Bayesian posterior probabilities values for clades of analyses (Figure 1). In this tree, the new genus is phylogenetically placed in the Leotiomycetes. This monophyletic group formed a close relationship with several genera, which are grouped in this class, e.g. *Vibrisseaflavovirens* and *Vibrisseatruncorum* (Vibrisseaceae), *Cudoniacircinans* and *Spathulariaflavida* (Cudoniaceae) that are grouped with the new genus in the same clade. Therefore, analysis of partial LSU and SSU nuc rDNA sequences placed the new genus in the Leotiomycetes. Additionally, the tree also supports the fact that the Helotiales is not monophyletic.

### Taxonomy

#### Blastosporium

Taxon classificationFungiLeotiomycetes

Z. F. Yu & H. Zheng gen. nov.

828280

##### Etymology.

Latin, *Blasto*-, referring to the blastic conidial ontogeny, + Latin, *sporium*, referring to the conidia.

##### Type species.

*Blastosporiumpersicolor* Z. F. Yu & H. Zheng

##### Diagnosis.

Characterised by mono- and polyblastic, integrated or discrete conidiogenous cells, solitary or blastocatenate, unicellular, obovoid, oblong, ellipsoidal, allantoid conidia (5–8 × 2.3–4.1 μm). Differs from the genus *Tetracladium* De Wild. by macronematous or semi-macronematous conidiophores and mono- and polyblastic conidiogenous cells.

##### Description.

Mycelium partly superficial and partly immersed, composed of branched, septate, smooth, hyaline hyphae. *Conidiophores* macronematous or semi-macronematous, erect or prostrate, smooth, hyaline, sometimes reduced to conidiogenous cells. *Conidiogenous cells* mono- and polyblastic, terminal, integrated or discrete, determinate, sometimes with sympodial elongations, smooth, hyaline. *Conidia* solitary or blastocatenate, acrogenous, unicellular, obovoid, oblong, ellipsoidal, allantoid, broad fusiform to irregular, smooth, hyaline.

##### Distribution.

China.

##### Notes.

*Blastosporium* is superficially similar to the genera, *Acaromyces* Boekhout et al. and *Meira* Boekhout et al. Their conidiophores are reduced to conidiogenous cells, which produce solitary or sometimes blastocatenate, unicellular, hyaline conidia by blastic conidial ontogeny. These genera are yeast-like hyphomycetes that have been connected phylogenetically with Exobasidiomycetidae (Ustilaginomycetes, Basidiomycota) (Boekhout et al. 2003, Seifert et al. 2011).

*Hyphozyma* de Hoog & M.T.Sm. also superficially resembles *Blastosporium*, but *Hyphozyma* is a typical yeast-like hyphomycete, characterised by undifferentiated conidiophores and conidia are unicellular, hyaline, solitary or produced in basipetal chains (de Hoog and Smith 1981, Seifert et al. 2011).

#### Blastosporium persicolor

Taxon classificationFungiLeotiomycetes

Z. F. Yu & H. Zheng sp. nov.

828281

Figure 2

##### Etymology.

Latin, *persicolor*, referring to the apricot colour of the colonies on PDA medium.

##### Description.

Colonies on CMA with 1–2 concentric rings slightly curled, entire at the margin, light orange-yellowish-pinkish colour. Reverse yellowish-orange. Mycelium partly superficial and partly immersed, composed of branched, septate, smooth-walled, creeping, 2.0–3.3 μm wide hyphae. *Conidiophores* macronematous or semi-macronematous, mononematous, erect or prostrate, straight or flexuous, unbranched or slightly branched, hyaline, smooth-walled, 35–14.4 × 1.8–3.5 μm. *Conidiogenous cells* mostly monoblastic, sometime polyblastic after several sympodial elongations, integrated or discrete, terminal or intercalary, 7.0–13.1× 2.6–3.3 μm, clavate or cylindrical, with a distinct or inconspicuous denticle at the conidiogenous loci. *Conidia* solitary or blastocatenate, acrogenous, obovoid, oblong, ellipsoidal, subcylindrical, allantoid, broad fusiform to irregular, slightly attenuated, truncate at the base or at the ends, unicellular, smooth, hyaline, 5–8 × 2.3–4.1 μm. Sexual form unknown.

**Figure 2.**
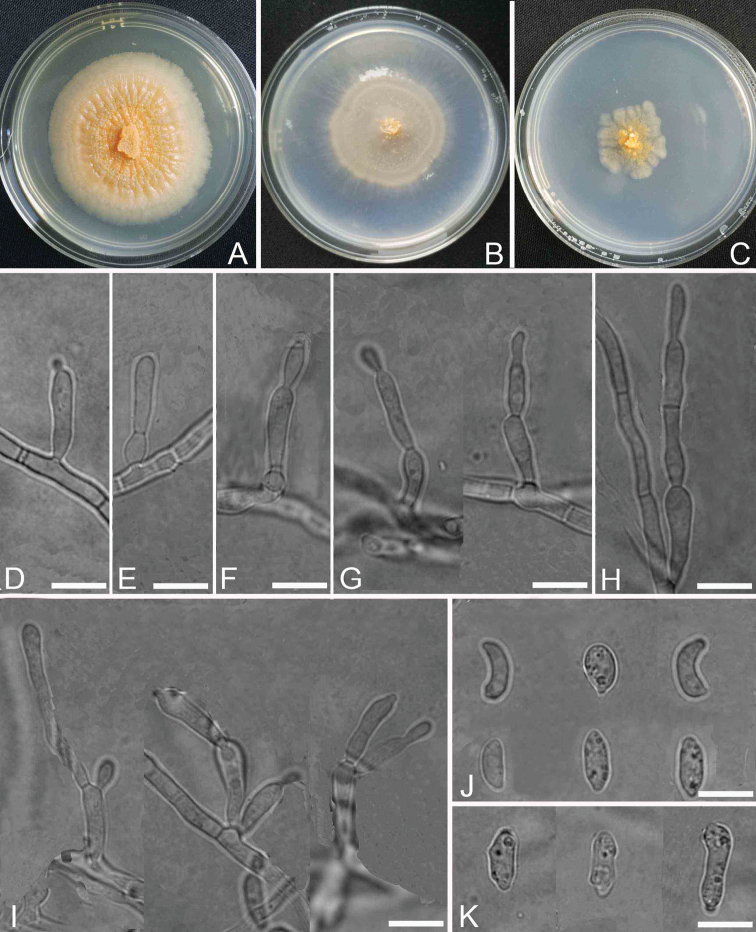
Cultures and anamorph of *Blastosporiumpersicolor* (YMF 1.05546). **A–C** Cultures (**A** on PDA**B** on CMA **C** on SNA) at 25 °C after 12 days **D–H** conidiophores and monoblastic conidiogenous cells **I** conidiophores and polyblastic conidiogenous cells **J, K** conidia (**J** one scar on conidia **K** multi-scars on conidia); Scale bar: 10 μm (**D–K**).

##### Culture characteristics.

(in darkness, at 25 °C after 10 d). Colonies attaining 1.5–1.7 cm diam. on PDA, 1.0–1.2 cm diam. on SNA, 1.5–1.7 cm on CMA. On PDA, colonies plicated, orange, reverse pale yellow, margin smooth and entire; sporulation abundant. On SNA, colonies flat, white to cream-coloured, flocculent, reverse white, growing slowly, sporulation abundant. The fungus does not grow at 35 °C on PDA, CMA and SNA.

##### Type.

**CHINA.** Xiamen, Fujian Province, 24°33'9.6"N, 117°55'7.4"E, 23 m alt., from mildewed tobacco (*Nicotianatabacum* L.) leaves, June 2018, Z.N. Zhang (dried slide YMFT 1.05546, holotype; ex-type YMF 1.05546).

## Discussion

To determine the phylogenetic placement of this species, *Blastosporiumpersicolor* was analysed with species from 7 classes, Leotiomycetes, Arthoniomycetes, Dothideomycetes, Eurotiomycetes, Orbiliomycetes, Pezizomycetes and Geoglossomycetes (Wang et al. 2006). By Bayesian analysis, the new genus was placed in the Helotiales, Leotiomycetes. In the tree, *B.persicolor* grouped with the *Cudonia*-*Spathularia* clade and *Vibrissea* clade, but the placement did not receive strong support. Therefore, we have temporarily designated this species as a new genus and family *incertae sedis*.

In the Helotiales, many genera, such as *Bulgaria* Fr. (Bulgariaceae), *Rutstroemia* P. Karst. (Rutstroemiaceae) and *Hegermila* Raitv. (Hyaloscyphaceae), were only observed as sexual morphs, but *Neofabraea* H.S. Jacks (Dermateaceae) and *Articulospora* Ingold (Helotiaceae) were observed as having asexual and sexual morphs (Chen et al. 2015, Wijayawardene et al. 2018, Wang et al. 2015a). In this study, we just observed the asexual morph of *B.persicolor*.

Based on ITS sequence data, *B.persicolor* is 88% similar to the genus *Tetracladium* De Wild. (*T.marchalianum* De Wild. as the type species), which was placed in the Helotiales and family incertae sedis. Moreover, *Blastosporium* shares some morphological features with *Tetracladium* as pale yellow and compact colonies and hyphae branched, septate and hyaline and both *Blastosporium* and *Tetracladium* sporulated abundantly on natural substrates (Sati et al. 2009, Wang et al. 2015b). However, *B.persicolor* is obviously distinct from the genus *Tetracladium* by the size and shape of conidia.

By molecular phylogeny analysis, *Blastosporium* belongs to the order Helotiales that currently contains 27 families (Wijayawardene et al. 2018). Moreover, members of the Helotiales cover a broad range of niches, such as plant pathogens, endophytes and aquatic hyphomycetes. *Blastosporiumpersicolor* was discovered from mildewed tobacco; therefore, it may be a plant pathogen.

## Supplementary Material

XML Treatment for Blastosporium

XML Treatment for Blastosporium persicolor
